# Overinterpretation and misreporting of prognostic factor studies in oncology: a systematic review

**DOI:** 10.1038/s41416-018-0305-5

**Published:** 2018-10-24

**Authors:** Emmanuelle Kempf, Jennifer A. de Beyer, Jonathan Cook, Jane Holmes, Seid Mohammed, Tri-Long Nguyên, Iveta Simera, Marialena Trivella, Douglas G. Altman, Sally Hopewell, Karel G. M. Moons, Raphael Porcher, Johannes B. Reitsma, Willi Sauerbrei, Gary S. Collins

**Affiliations:** 1 Centre for Statistics in Medicine, Nuffield Department of Orthopaedics, Rheumatology and Musculoskeletal Sciences, Botnar Research Centre, University of Oxford, Oxford, UK; 2 Department of Medical Oncology, Henri Mondor and Albert Chenevier Teaching Hospital, APHP, Créteil, France; 3 Laboratory UPRES EA2415, Biostatistics, Epidemiology, Clinical Research and Health Economics, University of Montpellier, Montpellier, France; 4 Nuffield Department of Medicine, Centre for Tropical Medicine and Global Health, University of Oxford, Oxford, UK; 5 Julius Center for Health Sciences and Primary Care, University Medical Center Utrecht, Utrecht University, Utrecht, The Netherlands; 6 Cochrane Netherlands, UMC Utrecht, Utrecht University, Utrecht, The Netherlands; 7 Department of Epidemiology, Hôtel Dieu Teaching Hospital, APHP, Paris, France; 8 Institute for Medical Biometry and Statistics, Faculty of Medicine and Medical Center, University of Freiburg, Freiburg, Germany; 9 NIHR Oxford Biomedical Research Centre, John Radcliffe Hospital, Oxford, OX3 9DU, UK

**Keywords:** Tumour biomarkers, Prognostic markers

## Abstract

**Background:**

Cancer prognostic biomarkers have shown disappointing clinical applicability. The objective of this study was to classify and estimate how study results are overinterpreted and misreported in prognostic factor studies in oncology.

**Methods:**

This systematic review focused on 17 oncology journals with an impact factor above 7. PubMed was searched for primary clinical studies published in 2015, evaluating prognostic factors. We developed a classification system, focusing on three domains: misleading reporting (selective, incomplete reporting, misreporting), misleading interpretation (unreliable statistical analysis, spin) and misleading extrapolation of the results (claiming irrelevant clinical applicability, ignoring uncertainty).

**Results:**

Our search identified 10,844 articles. The 98 studies included investigated a median of two prognostic factors (Q1–Q3, 1–7). The prognostic factors’ effects were selectively and incompletely reported in 35/98 and 24/98 full texts, respectively. Twenty-nine articles used linguistic spin in the form of strong statements. Linguistic spin rejecting non-significant results was found in 34 full-text results and 15 abstract results sections. One in five articles had discussion and/or abstract conclusions that were inconsistent with the study findings. Sixteen reports had discrepancies between their full-text and abstract conclusions.

**Conclusions:**

Our study provides evidence of frequent overinterpretation of findings of prognostic factor assessment in high-impact medical oncology journals.

## Introduction

Assessing the prognosis of patients with cancer is a key issue in clinical practice.^1^ In the era of precision or risk-based medicine, anticancer treatments are expected to be shaped by and tailored to the cancer’s aggression, among other prognostic factors (PFs). Cancer patients want to know their prognosis given their age, sex, tumour type, tumour stage or setting.^2–5^ A US study of 590 patients with advanced cancer found that 71% wanted to know their life expectancy.^6^ Telling advanced cancer patients their prognosis early on can improve their quality of life (QoL), anxiety, how well they assess their own life expectancy and their quality of death, and decrease the use of aggressive treatments near the end of life.^6–9^ For example, “dose-dense” regimens have been developed for the most aggressive tumour types, and “stop-and-go” therapeutic strategies may improve the QoL of patients with a good prognosis, while not favouring tumour growth.^10,11^ Disclosing a poor prognosis does not seem to impair the quality of the physician–patient relationship or patients’ hope.^2,6,12^

Many proposed PFs have disappointing clinical applicability, possibly because their effects are often overestimated in biomarker studies in oncology.^13,14^ For example, only 4 of the 28 published biological PFs for prostate cancer relapse after prostatectomy have been confirmed in independent studies.^15^ Prognosis research’s lack of reproducibility may be due to poor methodology,^16,17^ and poorly reported methods.^18–20^ A review of 50 tumour marker studies published in high-impact factor (IF) cancer journals found that only 36% clearly defined the patient outcomes of interest.^21,22^ Furthermore, the choice of appropriate statistical tools is a key component in assessing PFs in cancer patients, because their use and misuse have a considerable effect on the statistical significance of the study’s results.^23–25^

Reporting issues, such as spin, publication bias and selective reporting, not necessarily intended, is any strategy that leads to distorted study results, usually leading to more positive and significant results.^26^ Spin is the use of language to distort the interpretation of results and emphasise particular interpretations.^27,28^ How authors describe their results affects how readers interpret the findings. For example, a randomised clinical trial (RCT) showed that using words like “breakthrough” and “promising” to describe cancer drugs increased personal beliefs about the drugs’ effectiveness and the quality of the evidence presented.^29^ In another RCT of 300 specialised clinicians, Boutron et al.^30^ showed that a spin can convince clinicians that cancer treatment effects look more effective than what the study findings show.^30^

Reporting guidelines encourage authors to describe every detail of the methods and results when reporting their study. The REporting recommendations for tumour MARKer prognostic studies (REMARK) were published in 2005.^31,32^ Two years later, Kyzas et al.^33–35^ found severe publication issues and selective reporting in cancer PF studies. For example, among 1575 articles, they found that only 1.3% reported non-significant results without using spin or further analysis to make these results seem significant.^33^ Ten years later, is methodology still an issue in cancer PF studies, and what are the main strategies leading to study results’ inflation?

In this study, we estimate the type and the frequency of strategies distorting the presentation and the interpretation of the results in PF studies in oncology. We propose a classification system for the strategies used to misleadingly interpret and report PF studies. We then describe and assess the use of such strategies in a sample of oncology PF studies.

## Methods

### Search strategy and selection criteria

#### Ethical approval

This study did not require ethical approval as it was performed on available and published studies. The study was registered through the PROSPERO database (CRD42016039643).

#### Study selection

Studies were identified using the existing PubMed search filter “high specificity prognosis” (Clinical Queries/Prognosis/narrow, specific search). We combined the terms prognostic marker, PF, molecular marker AND malign*OR neoplasm*OR cancer AND survival, mortality, recurrence, prediction, outcome. We excluded reviews and meta-analyses, and added terms for commonly used biomarkers in oncology (Supplementary Table 1). We restricted our search to studies published during 2015 in oncology journals with the highest associated IFs, identified using Web of Science. We varied the IF cut-off threshold until a sample of approximately 100 studies was obtained. We excluded studies on haematology, studies that did not report PF assessment and studies of basic research performed in mice or cell lines. We classified articles into those investigating clinical factors and those investigating biomarkers as PFs. Any molecular abnormality (e.g. gene mutations) was considered as a biomarker. All outcomes were considered, regardless of treatments applied.

### Data analysis

#### Classification of misleading strategies

A classification scheme was developed of the strategies used by researchers that could mislead readers of PF studies. We based our classification on Fletcher and Black’s^36^ definition: “in writing an article [on PF studies], investigators have many opportunities to shape the impression their results produce in readers - by the statistical analyses they choose, the words they use to describe them, and the selection of results they choose to include in the article. This is so even though each analysis, word, and included piece of information might be legitimate in its own right”.

We classified strategies into three previously described categories: misleading reporting, misleading interpretation and misleading extrapolation of the results^37,38^ (Table 1). Each strategy refers to different steps within the study reporting and analysis. First, the authors are supposed to report the raw data of their study, exhaustively and as planned in the Methods section. We defined whatever deviates as “Misleading reporting”. Second, the authors are welcome to interpret their study results, which implies a deliberate implementation of statistical tests and a personal opinion of the meaning of the results. Finally, the authors are supposed to mention what research and clinical implications their study results might have in the future. We classified irrelevant and overoptimistic author’ suggestions as “Misleading extrapolation”. Each of these strategies were described in a recent systematic review on the use of spin in biomedical research published by Chiu and colleagues.^39^ We collected strategies described in similar reviews on misleading reporting, misleading interpretation and misleading extrapolation in therapeutic RCTs,^27,40^ diagnostic test accuracy studies,^41,42^ non-randomised therapeutic trials^37^ and in systematic reviews and meta-analyses.^38^ An arbitrary sample of 50 articles that assessed a PF for cancer patients were reviewed to identify missing strategies. Once we had a list of possible strategies, we identified the different approaches by which study results are most frequently distorted in PF oncology studies.^17^ A distinction was made between abstracts and full text, as many readers use abstracts as their sole source of scientific information or to identify articles to read.

**Table 1:** Classification of the misleading strategies used by authors when presenting prognostic factor studies in oncology

Misleading reporting	Misleading interpretation	Misleading extrapolation
•Selective reporting *Pre-planned analyses are not reported*	•Unreliable statistical analysis *Inappropriate statistical strategy*	•Ignoring uncertainty *Unjustified strong conclusions*
•Incomplete reporting *Analyses are partially reported*	•Spin *Rhetorical and formal strategy to inflate study results*	•Claiming irrelevant clinical applicability *Inappropriate generalisations*

#### Misleading reporting

We identified two ways that authors can mislead the reader by withholding information: selective reporting and incomplete reporting. An author selectively reports when they do not present the results of all of their planned analyses, instead choosing to report only a subset of results. This selective reporting results in discrepancies between the planned analyses presented in the Methods section and the reported results. An author incompletely reports when they leave out essential information when reporting a particular study result. For example, an author might report a PF effect using an adjusted hazard ratio, but not report its precision with a 95% confidence interval or *p* value.

#### Misleading interpretation

We identified two strategies that mislead the reader through the analysis and the interpretation of the data: choosing an unreliable statistical analysis strategy and spin. Some unreliable statistical analysis strategies are more likely to give them statistical significance, but greater risk of a false-positive result. For example, the most reliable way to assess a potential PF’s effect is using a multivariable model and, when performing subgroup analysis, using an interaction test.^31^ Articles might instead report only whether the log-rank test *p* value is <0.05 or report only unadjusted models. Running *ad hoc* analyses, like unplanned subgroup analyses, and multiple statistical analyses also increases the chance of falsely reaching statistical significance. So too does handling continuous variables in multiple ways (e.g. dichotomising using different thresholds), as it increases the number of statistical analyses and therefore the probability of finding a significant association by chance. Spin is a rhetorical strategy used to exaggerate study results, using language to highlight positive results (those agreeing with the authors’ hypothesis) and suppress negative results (those showing no effect or disagreeing with the authors’ hypothesis). Subjective comments that spin the quantitative value of a study’s findings can also be used to mislead interpretation. Examples of strong statements that spin a study’s findings are words that imply a value judgement, like “efficient” or “valuable”, or words that imply a causal inference between the PF and outcome. Report syntax may reject the non-significance of the results by referring to expressions like “trending toward significance” and “almost achieving significance”. Lack of consistency between the study findings and the report title, abstract and full-text conclusions may distort the readers’ interpretation of study results.

#### Misleading extrapolation

We identified two ways likely to mislead readers by extrapolating from the results to inappropriate generalisations: ignoring uncertainty and claiming irrelevant clinical applicability. Conclusions may disregard the uncertainty that is inherent in any study, summarising the study findings as if they are an established fact. The external validation would ideally be prospective and performed by independent investigators. Claiming PF clinical applicability might lack relevance. For example, conclusions may try to increase the generalisability of the study results by involving broader clinical settings than those studied or by using inconsistent surrogate outcomes.

#### Data extraction

We developed and pilot-tested a data extraction form (available on request) based on our misleading strategies classification system. Duplicate extraction was performed. The first extractor (E.K.) is a medical oncologist and the second extraction was carried out by randomly allocating articles to one of seven biostatisticians and researchers (J.A.dB., J.C., J.H., S.M., T.-L.N., I.S., M.T.). Any discrepancies were discussed until agreement was reached.

The general characteristics of each study were extracted from the report: academic status and scientific background of the first author, funding source (non-profit, for-profit, both, not reported), disclosure of authors’ conflicts of interest (COIs), whether the original study that recruited the patients was randomised or non-randomised, mention of adherence to the REMARK guideline, whether the PF was defined when the patients were included in the study (prospective assessment) or was assessed retrospectively using existing data or stored human material (retrospective), the number and type of patient outcomes, number and type of PFs, sample size, number of events for each outcome and length of patient follow-up.

We also extracted items to assess the use of misleading strategies. We assessed the statistical methods used to determine the PF effect, any variable selection procedures, whether any subgroup analysis was pre-specified, how many statistical associations between the PFs and outcomes were planned in the Methods section, how many PF–outcome associations were reported in the results section, the type of statistical tests supporting these associations (e.g. log-rank test, multivariable model), whether the discussion mentioned studies that agreed or disagreed with the results, the use of linguistic spin (e.g. inferring a causal relationship between the PF and outcome) and whether the non-significance of any results was rejected. We evaluated whether the title, abstract conclusion and full-text conclusion suggested that the PFs had clinical applicability, in which setting and whether this conclusion agreed with the study results.

## Results

### Study selection

**Fig. 1:**
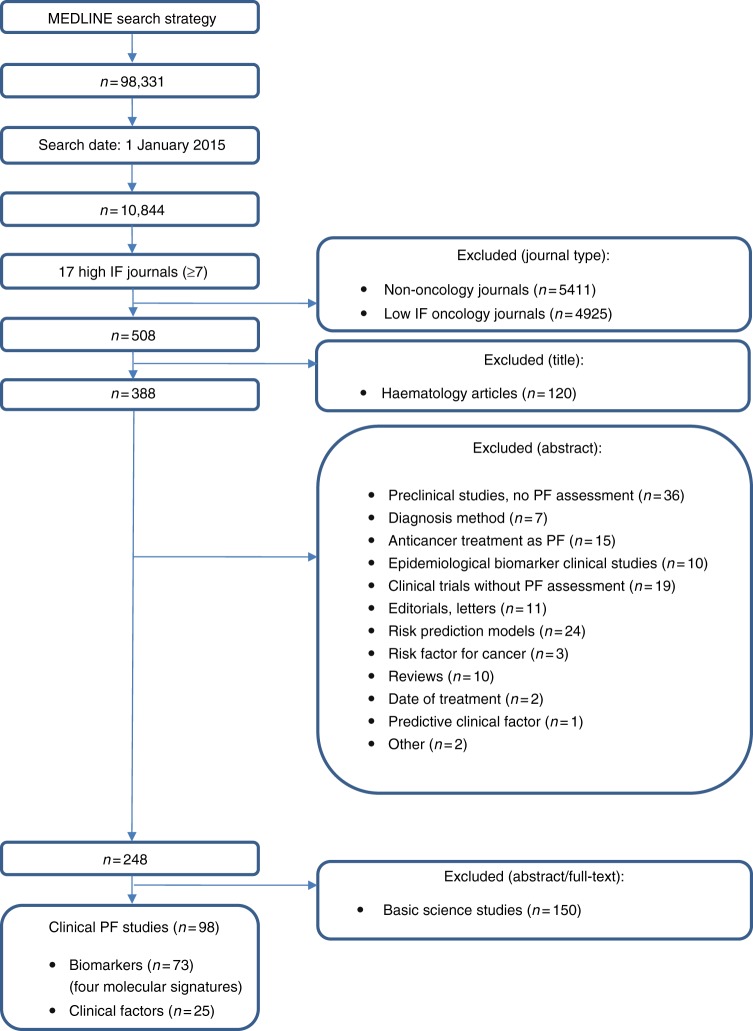
Flow chart of the study selection

The study selection is summarised in Fig. 1. Our search string identified 10,844 articles. We excluded 5411 articles published in non-oncology journals and 4925 reports published in oncology journals with an IF <7. Supplementary Table 2 lists the 19 targeted oncology journals. We excluded 120 haematological articles, 140 articles that did not report PF assessment, and 150 basic biology studies. The remaining 98 papers were eligible and included in the review.

### Study characteristics

Of the 98 reports, 73 focused on biomarkers and 25 on clinical factors, while 54 included data from observational studies and 44 from randomised trials. The included studies are listed in Supplementary Table 3. The median IF was 8.2. Journal websites did not make cited online supplemental files available for nine reports.

Eighty-eight studies reported their funding sources, of which 31 received industry funding. Eight-nine studies included a COI section in their report, but only 48 reported at least one COI. The scientific background of the first author was reported in 50 articles: 39 clinicians, 7 biologists and 4 epidemiologists. A statistician or epidemiologist was reported among the co-authors in 26 studies.

The PF was assessed prospectively in 8 of the 54 observational and 18 of the 44 interventional reports. Thirty of the observational studies had both retrospective patient follow-up and PF assessment. Eighteen studies used two or more independent patient populations to validate the PF association. Twelve reports mentioned the REMARK reporting guideline.

The median sample size was 259 patients (first quartile (Q1)–third quartile (Q3), 102–904). Fifty-seven studies reported the number of events for the main PF, with a median of 128 events (Q1–Q3, 29–338). Sixty-two studies reported the patient follow-up time, with a median of 56.4 months (Q1–Q3, 31.5–80.3). The median number of PFs assessed per study was 2 (Q1–Q3, 1–7), and the median number of outcomes was 2 (Q1–Q3, 2–3). Overall survival was used to assess the PF effect in 66 reports.

Thirty-seven reports used two or more multivariable models to assess the PF effect (as defined by the type of the adjustment variables), and 21 studies did not adjust their analyses. Eighty-eight articles did not explain how missing data were addressed. Seventy-one studies categorised continuous variables, and 54 among them did not describe how continuous variables were handled. Thirty-four reports used multiple definitions for the PF within the same study, such as assessing the PF effect as both a continuous and dichotomised variable, or running multiple analyses with different categorisation thresholds.

### Use of misleading strategies

Tables 2, 3, and 4 summarise the frequency of each misleading strategy (Supplementary Table 4 displays the full data extracted).

**Table 2:** Classification of misleading reporting strategies used by authors when presenting prognostic factor studies in oncology, and frequency of each strategy in a sample of 98 prognostic factor studies published in oncology journals with an impact factor of seven or greater

Misleading reporting strategy	Places within a prognostic factor oncology study report where this strategy can occur	Test to check whether this strategy was used	Number of studies using this strategy	
			Main text	Abstract
Selective reporting	Difference between the numbers of outcomes and prognostic factors pre-specified in Methods section and reported in Results section	The prognostic factor effect for a specific outcome or a specific prognostic factor is missing	35	34
	Difference between the numbers of subgroup and subpopulation criteria pre-specified in Methods section and reported in Results section	The prognostic factor effect for a specific subgroup or a subpopulation is missing	5	Not assessed
	Inconsistent use of statistics across all prognostic factor–outcome associations, when more than one prognostic factor effects are assessed	Some PF effects are reported within a multivariable model, whereas others are reported within a univariate analysis	24	41
	Incomplete reporting of subgroup analysis results for the prognostic factor effect	For any subgroup analysis reported, whether pre-specified or not, an interaction test *p* value is not reported	35 (out of 90 studies assessing more than one prognostic factor effect)	42 (out of 90 studies assessing more than one prognostic factor effect)
Incomplete reporting	Incomplete reporting of the main analysis results for the prognostic factor effect	Only adjusted hazard ratios are reported for the prognostic factor effect, not 95% confidence intervals or *p* values; OR Only a *p* value is reported for the prognostic factor effect, not adjusted hazard ratios; OR No statistical results are reported	24	41
	Incomplete reporting of subgroup analysis results for the prognostic factor effect	For any subgroup analysis reported, whether pre-specified or not, an interaction test *p* value is not reported	29 (out of 51 studies reporting a subgroup analysis in the main text)	23 (out of 28 studies reporting a subgroup analysis in the abstract)

Of the 57 abstracts presenting unadjusted prognostic factor effects, 23 reported were related to non-significant findings after adjustment in the full text

**Table 3:** Classification of misleading interpretation strategies used by authors when presenting prognostic factor studies in oncology, and frequency of each strategy in a sample of 98 prognostic factor studies published in oncology journals with an impact factor of seven or greater

Misleading interpretation strategy	Places within a prognostic factor oncology study report where this strategy can occur	Test to check whether this strategy was used	Number of studies using this strategy	
			Main text	Abstract
Statistical issue	Reported significance of the prognostic factor effect is based on a subgroup analysis that was not pre-specified	No subgroup analysis was pre-specified in the Methods section	34 (out of 51 studies that reported a subgroup analysis)	34 (out of 51 studies that reported a subgroup analysis)
	Reported significance of the prognostic factor effect is based on a subpopulation analysis that was not pre-specified	No subpopulation analysis was pre-specified in the Methods section	21 (out of 31 studies that reported a subpopulation analysis)	5 (out of 6 studies that reported a subpopulation analysis)
	Reported significance of the prognostic factor effect in a subgroup analysis is not based on the *p* value of the interaction test	The prognostic factor effect across subgroups is not reported with a *p* value from an interaction test	26 (out of 51 studies that reported a subgroup analysis)	4 (out of 28 studies that reported a subgroup analysis)
Spin	Use of strong statements	The prognostic factor effect is described with a value judgement like “efficient”; OR	12 (mentioned in the Results section)	10 (mentioned in the Results section)
		A causal inference between the prognostic factor and outcome is mentioned	29 (mentioned in the Discussion section)	29 (mentioned in the Conclusion section)
	OR Reject the non-significance of a prognostic factor effect	The prognostic factor effect is said to be significant, although the 95% confidence interval of the adjusted odds ratio crosses 1; OR Words like “trend” or “borderline significance” are used	34 (mentioned in the Results section) 28 (mentioned in the Discussion section)	15 (mentioned in the Results section) 17 (mentioned in the Conclusion section)
	Use of any type of linguistic spin	The PF effect is reported with strong statement OR its non-significance is rejected	46 (mentioned in the Results section) 57 (mentioned in the Discussion section)	25 (mentioned in the Results section) 46 (mentioned in the Conclusion section)
	Title is inconsistent with the study results	Title is supportive of prognostic factor significance, despite the study reporting a non-significant effect	10 (out of 30 titles supportive of a prognostic factor effect)	Not applicable
	Discussion and/or abstract conclusions are inconsistent with the study findings		20	21
	Discrepancies between the full-text (discussion) and abstract (conclusion) explanations of the study findings	The discussion is consistent with the study findings, whereas the abstract conclusion is not [+/−]; OR The discussion is not consistent with the study findings, whereas the abstract conclusion is [−/+]		16 8 [+/−] 8 [−/+]
	No mention of the study’s limitations in the discussion		34	Not applicable
	Conclusion focuses solely on significant results	If at least one non-significant PF–outcome association in a multivariable model is reported in the results, this result is not mentioned in the conclusion	41 out of 50 studies that reported at least one non-significant PF–outcome association	Not assessed
	Main results are reported in an online supplemental file	PF–outcome associations are reported in supplemental files	54	Not applicable
	Spin in tables or figures	Non-significant *p* values adjusted for multiple comparisons are written below the table, whereas significant unadjusted *p* values are highly visible	32	Not applicable

**Table 4:** Classification of misleading extrapolation strategies used by authors when presenting prognostic factor studies in oncology, and frequency of each strategy in a sample of 98 prognostic factor studies published in oncology journals with an impact factor of seven or greater

Misleading extrapolation strategy	Places within a prognostic factor oncology study report where this strategy can occur	Test to check whether this strategy was used	Number of studies using this strategy	
			Main text	Abstract
Irrelevant clinical applicability	Claiming prognostic factor clinical applicability even though the study used surrogate outcomes that have not yet been validated	If the prognostic factor is reported to have clinical applicability, when using an outcome other than overall survival or quality of life	16 (out of 32 studies using outcomes other than overall survival of quality of life)	25 (out of 32 studies using outcomes other than overall survival of quality of life)
	Claiming prognostic factor clinical applicability in a different or unclear clinical setting	If the prognostic factor is reported to have clinical applicability in a patient population which is not clearly described, or different from the study population	27 (out of 44 studies reporting external clinical applicability)	18 (out of 44 studies reporting external clinical applicability)
Ignoring uncertainty in the results	No mention of uncertainty about the study results	Eternal present tense is used in the conclusion without any verbs as “may” or “could”, nor any words as “likely to” or “may be	59	75
	No mention of further study needed to assess PF validity	No mention of the need for external validation using a different data set	57	91

Among misleading reporting strategies, selective and incomplete reporting were found in 90 and 55 studies, respectively. The PF effect was selectively reported in 35 of the 98 main texts. Out of 50 studies reporting at least one non-significant PF–outcome association, 41 conclusions focused solely on significant results. Among misleading interpretation strategies, unreliable statistical analysis was used in 49 studies, and linguistic spin was found in 75 reports. Authors used spin to reject non-significant results in 34 main texts. Among misleading extrapolation strategies, clinical applicability was found irrelevant in 35 out of 55 reports and the uncertainty of the results was ignored in 48 studies.

## Discussion

We created a classification system of the common strategies likely to mislead readers, finding seven general strategies across three domains (misleading reporting, misleading interpretation, and misleading extrapolation). Each of these strategies could also be used in multiples places within a report. We assessed how often each of these strategies was used in 98 articles published in high IF oncology journals. Most of the PFs studied were biomarkers and their effect was likely to be assessed as post hoc analyses of previous studies, often not conducted by statisticians.

We found misleading reporting strategies to be widely used in PF studies in oncology. Selective reporting can affect outcome and statistical analysis reporting, influencing how readers interpret study findings.^43,44^ Thirty-five studies selectively reported the PF effect, either not reporting all outcomes or not reporting all PFs. This result agrees with studies of other types of research: half of all RCTs report at least one outcome that either does not appear in the study protocol or is omitted or is changed when compared to their protocols.^26^ Statistically significant and positive results in RCTs are more likely to be reported than non-significant results,^26^ which is consistent with our findings. Among 50 studies that used a multivariable model and found at least one non-significant PF effect, we found 41 conclusions focused solely on the significant results. Vera-Badillo et al.^45^ showed that half of cancer RCTs with non-significant results reported the primary outcome by favouring the intervention’s statistical significance.^45^ Two-thirds of RCTs reported drug-related toxicity in an irrelevant way, especially when the experimental treatment showed better outcomes than the control.^45,46^

Misleading interpretation strategies were also widespread in our sample of studies. Only eight PF studies in our review were prospective studies that prospectively assessed the PF of interest. Looking for PFs in an existing patient data set can result in “HARKing” (hypothesising after the results are known), if clinical data are recycled for post hoc analyses that are reported as a priori hypotheses.^47^ This strategy leads to assessing multiple outcomes, with multiple models, in multiple subpopulations, while running multiple statistical analyses, which all increases the chance that statistical significance will be reached by chance. Unplanned analyses to “cherry-pick” were rife, with 45 out of 61 statistical analyses performed in subpopulations that were not pre-specified in the methods, for example.^44,48,49^ These strategies link with incomplete reporting, with 31 of the 56 studies that examined multiple PFs reporting the results of different statistical tests for each PF, presumably to avoid reporting non-significant multivariable results. Poor statistical methodology choices were thus common in the studies we reviewed, as has generally been found in the research on prognosis research literature.^35^ Twenty-one of the studies that we analysed displayed a Conclusion content which was inconsistent with the full-text results. The same observation was made in a review of diagnostic test accuracy studies, for both imaging and biomolecular tests separately.^41^ In this review, authors highlighted a form of potential overinterpretation in 99% of the studies (including the use of inappropriate statistical tools and not pre-specified subgroups in the Methods section).

Structured displays may help authors to report the necessary information transparently and completely, avoiding the incomplete and selective reporting that we have noted. Altman et al.^31^ developed a two-part structured display for reporting study profiles, which was used in a study of the reporting of prognostic studies^21^ and was extended by Winzer et al.^50^ The first part gives details about how the marker of interest was handled in the analysis and which other variables were available. Patient population, inclusion and exclusion criteria, and the number of eligible patients and events for each outcome in the full data set are also reported. To help the reader understand the multiplicity of analyses and better assess the results, the second part of the proposed profile gives an overview of all analyses, including early steps conducted in an initial data analysis and check of important assumptions.

We found linguistic spin using strong statements or rejecting non-significant results in 57 report discussions and in 46 abstract conclusions, which is consistent with Kyzas et al.'s^33^ findings. Although linguistic spin is to some extent an expected part of scientific writing,^36,51^ it can have serious consequences. When linguistic spin is used to report non-significant RCT results, how readers interpret the study findings becomes distorted.^27,52^ The press is also more likely to report health research findings if they are presented with linguistic spin.^53,54^

The reviewed studies also frequently extrapolated their findings beyond their actual results. Fifty-seven reports did not mention the need for any validation study to confirm the prognostic value of their PF. This result is consistent with the literature: half of the observational studies assessing a medical intervention recommend its application in clinical practice without mentioning the need for an RCT.^55^ Out of 55 studies referring to external validity, 35 reports extrapolated the clinical applicability of their PF to a different or an unclear setting, which agrees with the results of a previous review of 108 biomarker studies, in which 56% of the reports exaggerated the related clinical applicability.^56^ Half of the studies that used a surrogate outcome that has not yet been validated still claimed clinical applicability for their PF (16/32 studies). As readers might skim a published report’s results and focus on its conclusions, clinical recommendations should be consistent with the study’s clinical setting so as not to mislead the reader.

The poor reporting and unreliable methodology that we have highlighted here may explain why biomarker study findings lack reproducibility.^57^ This systematic review might give a thoughtful perspective to biomedical journal readers and help them to understand how prognosis biomarker research is a delicate process. This article might raise the awareness of scientists who are keen on identifying new cancer PF about the need of a specialised expertise in methodology and statistics in order to publish clinically relevant and robust results. A poorly reported study is difficult to reproduce, as key details are missing, and cannot be judged properly at peer review or post publication. More widespread use of the REMARK guideline, BRISQ criteria^58^ and type profile, as well as the TRIPOD statement, would improve the quality of published prognosis research.^59^ Selective reporting could also be offset by giving regulatory agencies access to a summary of the pivotal results of prognostic studies, as is done for trials.^60,61^ Journals can also help by encouraging authors to publish non-significant PF effects.^62^ Mandatory preregistration of standardised prognostic studies, as with RCTs, could also help to offset these reporting issues.^63–65^ The methodology used in prognostic studies could be improved by involving a statistician or epidemiologist in the design of studies dedicated to PF assessment.^66,67^ Scientific societies, such as ASCO or NCCN, are key stakeholders in publishing guideline regarding the use of PFs in clinical practice.

We acknowledge several limitations of this study. We used journal IF to select our studies. High IF journals are assumed to have good quality peer review and editorial processes, so these studies may be of better reporting quality than all similar studies. However, high IF journals also tend to publish significant, positive results, so these studies may be at great risk of containing spin and overinterpretation.^56,68–70^ Some of the extracted items required the extractor to make a subjective decision. For example, whether words such as “novel” and “perfect association” constitute linguistic spin is a subjective judgement. Extractors also distinguished between PFs and confounding variables in multivariable models, which is again a subjective decision. We did not address the impact of misleading reporting and interpretation strategies on the use of PFs in routine clinical practice among clinicians. Our findings cannot be generalised beyond oncology.

Our study has several strengths. This study involved researchers specialised in clinical oncology and in prognosis research. We based our classification system on a framework that has been used to study misleading strategies in reporting and interpretation of studies in several other health research areas.^37,38^ Most of the extracted items did not require a subjective judgement by the extractor, such as the type and number of statistical tests used.

Prognosis research in oncology is often biomarker-driven. Although much innovative work has been done in cancer biomarker research, the clinical applicability of many identified biomarkers might still look a bit disappointing. We found that cancer prognosis research is likely to use some unreliable methodology and misleading reporting. Some conclusions drawn might lack strong clinical relevance and pure consistency with the numerical study findings. For example, we found a few discrepancies between the conclusions presented in the full text and abstract. Adherence to international, such as the REMARK, guideline in primary PF studies could improve the reporting and critical appraisal of prognosis research in cancer. Future biomarker studies will be based on a better, clearer evidence base, increasing the chance of clinical applicability.

## Electronic supplementary material

Supplementary Table 1

Supplementary Table 2

Supplementary Table 3

Supplementary Information
