# Supplementary material for: Overinterpretation and misreporting of prognostic factor studies in oncology: a systematic review
Source: Br J Cancer. 2018 Oct 24;119(10):1288–96. doi: 10.1038/s41416-018-0305-5 (PMC6251031; doi:10.1038/s41416-018-0305-5)
Supplement: Supplementary file 1 — Supplementary Table 1 [file 41416_2018_305_MOESM1_ESM.pdf]

Supplementary Table 1 – Final search algorithm

(Prognosis/Narrow[filter]) AND (((((tumour marker OR prognostic marker OR progn* marker OR prognostic factor OR progn* factor OR molecular marker OR tumour protein OR p53 OR VEGF OR MVD OR K-ras OR cmyc OR Cathepsin OR cox OR HIF OR EGFR OR ki-67 OR c-erbB-2 OR BMM OR DNA ploidy) AND (malign* OR neoplasm* OR cancer OR tumour) AND (survival OR mortality OR recurrence OR relapse OR outcome OR predict*) NOT (review OR metaanalysis))))).
