# Supplementary material for: Overinterpretation and misreporting of prognostic factor studies in oncology: a systematic review
Source: Br J Cancer. 2018 Oct 24;119(10):1288–96. doi: 10.1038/s41416-018-0305-5 (PMC6251031; doi:10.1038/s41416-018-0305-5)
Supplement: Supplementary file 2 — Supplementary Table 2 [file 41416_2018_305_MOESM2_ESM.pdf]

Supplementary Table 2. Selected oncology journals indexed by their impact factor

Journal Title	ISSN	2014 Total Cites	2014/2015 Impact Factor	5-Year Impact Factor	Immediacy Index	2014 Articles	Cited Half-life	*Eigenfactor®* Score	*Article Influence®* Score
CA: A Cancer Journal for Clinicians	0007-9235	18594	144.800	131.810	35.923	26	3.5	0.06273	39.508
Nature Reviews Cancer	1474-175X	39868	37.400	44.335	4.377	61	7.4	0.10009	18.217
Lancet Oncology	1470-2045	24861	24.690	26.239	6.243	169	4.2	0.10174	10.011
Cancer Cell	1535-6108	27283	23.523	27.252	4.707	116	5.7	0.10680	13.240
Cancer Discovery	2159-8274	4605	19.453	20.259	5.761	92	2.1	0.03146	9.856
Journal of Clinical Oncology	0732-183X	133258	18.443	16.971	5.496	464	6.4	0.34681	6.158
Nature Reviews Clinical Oncology	1759-4774	4462	14.180	14.916	3.827	52	3.4	0.02520	5.569
JNCI: Journal of the National Cancer Institute	0027-8874	36458	12.583	13.584	2.173	173	>10.0	0.06275	5.811
Blood	0006-4971	150854	10.452	9.57	2.42	853	7.10	0.37425	3.63
Leukemia	0887-6924	20905	10.431	9.158	4.226	212	5.9	0.05738	3.425
Seminars in Cancer Biology	1044-579X	5094	9.330	8.105	3.039	51	6.7	0.01096	2.697
Cancer Research	0008-5472	142659	9.329	9.115	1.406	679	9.1	0.21155	3.126

Journal Title	ISSN	2014 Total Cites	2014/2015 Impact Factor	5-Year Impact Factor	Immediacy Index	2014 Articles	Cited Half-life	*Eigenfactor*® Score	*Article Influence*® Score
Clinical Cancer Research	1078-0432	72155	8.722	8.531	1.854	609	6.6	0.16125	2.824
Oncogene	0950-9232	64071	8.459	7.632	1.963	572	8.4	0.10362	2.627
BBA Reviews on Cancer	0304-419X	3964	7.845	8.811	1.512	82	5.3	0.01037	2.688
Cancer Treatment Reviews	0305-7372	5719	7.588	6.611	2.786	126	5.0	0.01388	1.888
The Journal of Pathology	0022-3417	15629	7.429	6.941	1.791	148	7.7	0.03163	2.400
Cancer and Metastasis [illegible]	0167-7659	5649	7.234	9.312	0.716	74	7.0	0.01105	2.784
Annals of Oncology	0923-7534	26807	7.040	6.885	1.736	345	4.9	0.08077	2.316
