# Supplementary material for: Overinterpretation and misreporting of prognostic factor studies in oncology: a systematic review
Source: Br J Cancer. 2018 Oct 24;119(10):1288–96. doi: 10.1038/s41416-018-0305-5 (PMC6251031; doi:10.1038/s41416-018-0305-5)
Supplement: Supplementary file 3 — Supplementary Table 3 [file 41416_2018_305_MOESM3_ESM.pdf]

Supplementary Table 3. Bibliographic details of the 98 articles included in this study

First author	Last author	Journal	2013 Impact Factor	2014 Total Cites	Title (beginning of)
**BIOMARKERS**					
Bellmunt	Signoretti	Ann Oncol	6.578	26807	Association of PD-L1 expression on tumor-infiltrating mononuclear cells
Cierna	Babal	Ann Oncol	6.578	26807	Prognostic value of programmed-death-1 receptor (PD-1) and its ligand 1
Curigliano	Viale	Ann Oncol	6.578	26807	Risk of subsequent in situ and invasive breast cancer
Davis	Martin	Ann Oncol	6.578	26807	EVERSUN: a phase 2 trial of alternating sunitinib and everolimus
Dieci	Goubar	Ann Oncol	6.578	26807	Prognostic and predictive value of tumor-infiltrating lymphocytes
Grunwald	Gauler	Ann Oncol	6.578	26807	TEMHEAD: a single-arm multicentre phase II study of temsirolimus
Kang	Kim	Ann Oncol	6.578	26807	Prognostic value of tumor-infiltrating lymphocytes in Epstein–Barr virus
Kang	Kudo	Ann Oncol	6.578	26807	Randomized phase II study of axitinib versus placebo plus best supportive care
Kote-Jarai	Eeles	Ann Oncol	6.578	26807	Prevalence of the HOXB13 G84E germline mutation in British men
Massarelli	Papadimitrakopoulou	Ann Oncol	6.578	26807	Phase II trial of everolimus and erlotinib in patients
Michels	Kroemer	Ann Oncol	6.578	26807	Negative prognostic value of high levels of intracellular poly(ADP-ribose)
Montagna	Colleoni	Ann Oncol	6.578	26807	Changes in PgR and Ki-67 in residual tumour and outcome
Pruneri	Viale	Ann Oncol	6.578	26807	Clinical validity of tumor-infiltrating lymphocytes analysis
Rulli	Garassino	Ann Oncol	6.578	26807	Value of KRAS as prognostic or predictive marker in NSCLC
Sclafani	Valeri	Ann Oncol	6.578	26807	Prognostic role of the LCS6 KRAS variant in locally advanced rectal cancer
Sotelo	Diaz-Rubio	Ann Oncol	6.578	26807	Role of circulating tumor cells as prognostic marker
Speirs	Coombes	Ann Oncol	6.578	26807	Prognostic and predictive value of ER(31 and ER(32
Sunakawa	Lenz	Ann Oncol	6.578	26807	Association of variants in genes encoding for macrophage-related functions

Vansteenkiste	O'Byrne	Ann Oncol	6.578	26807	Cilengitide combined with cetuximab and platinum-based chemotherapy
Barrera	Arrieta	Ann Oncol	6.578	26807	Cytokine profile determined by data-mining analysis set into clusters
Tobin	Lindstrom	Ann Oncol	6.578	26807	Molecular subtype and tumor characteristics of breast cancer metastases
Grenader	Bridgewater	Ann Oncol	6.578	26807	Derived neutrophil lymphocyte ratio may predict benefit from cisplatin
Joseph	Choudhury	Ann Oncol	6.578	26807	Pre-treatment lymphocytopaenia is an adverse prognostic biomarker
Lorente	de Bono	Ann Oncol	6.578	26807	Baseline neutrophil–lymphocyte ratio (NLR) is associated with survival
van Soest	de Wit	Ann Oncol	6.578	26807	Neutrophil-to-lymphocyte ratio as a prognostic biomarker for men
Koguchi	Bahjat	Cancer Res	9.284	142 659	Serum immunoregulatory proteins as predictors
McDaniel	Tomlins	Cancer Res	9.284	142 659	Genomic profiling of penile squamous cell
Abdel-Fatah	Chan	Clin Cancer Res	8.193	72 155	HAGE in triple-negative breast cancer
Apellaniz-Ruiz	Rodriguez-Antona	Clin Cancer Res	8.193	72 155	Whole-exome sequencing reveals defective
Bell	Chakravarti	Clin Cancer Res	8.193	72 155	SMARCA4/BRG1 is a novel prognostic biomarker predictive of cisplatin-based
Ben-Josef	Williams	Clin Cancer Res	8.193	72 155	Glycogen synthase kinase 3 beta predicts survival
Bohanes	Lenz	Clin Cancer Res	8.193	72 155	Pharmacogenetic analysis of INT 0144 trial:
Burdelski	Steurer	Clin Cancer Res	8.193	72 155	Cytoplasmic accumulation of sequestosome 1
Cushman	Nixon	Clin Cancer Res	8.193	72 155	Gene expression markers of efficacy and
Dahlstrom	Sturgis1	Clin Cancer Res	8.193	72 155	HPV serum antibodies as predictors of survival
Deckers	van Engeland	Clin Cancer Res	8.193	72 155	Promoter methylation of CDO1 identifies clear-
Donnem	Busund	Clin Cancer Res	8.193	72 155	Stromal CD8þ T-cell density—a promising
Gradishar	Haluska	Clin Cancer Res	8.193	72 155	Clinical and translational results of a phase II
Hong	Nemunaitis	Clin Cancer Res	8.193	72 155	Phase I dose-escalation study of the multikinase
Maréchal	van Laethem	Clin Cancer Res	8.193	72 155	Sonic hedgehog and Gli1 expression predict
Paoletti	Hayes	Clin Cancer Res	8.193	72 155	Significance of circulating tumor cells in
Pierga	Viens	Clin Cancer Res	8.193	72 155	Pathological response and circulating tumor cell

Santiago-Walker	Martin	Clin Cancer Res	8.193	72 155	Correlation of BRAF mutation status in
Sinicrope	Alberts	Clin Cancer Res	8.193	72 155	Analysis of molecular markers by anatomic tumor
Vassilakopoulou	Psyrri	Clin Cancer Res	8.193	72 155	Evaluation of PD-L1 expression and associated
Zhang	Wang	Clin Cancer Res	8.193	72 155	Direct serum assay for cell-free Bmi-1 mRNA and
Hingorani	Skapek	Clin Cancer Res	8.193	72 155	Clinical application of prognostic gene
O'Brien	Timms	Clin Cancer Res	8.193	72 155	Serum CA19-9 is significantly upregulated up to 2
André	de Gramont	J Clin Oncol	17.879	133 258	Adjuvant fluorouracil, leucovorin, and oxaliplatin in stage
Catenacci	Kindler	J Clin Oncol	17.879	133 258	Randomized phase Ib/II study of gemcitabine plus placebo
Joensuu	Emile	J Clin Oncol	17.879	133 258	KIT and PDGFRA mutations and the risk of GI stromal
Kopetz	Saltz	J Clin Oncol	17.879	133 258	Phase II pilot study of vemurafenib in patients with
Mistry	Tabori	J Clin Oncol	17.879	133 258	BRAF mutation and CDKN2A deletion define a
Rosenthal	Bonner	J Clin Oncol	17.879	133 258	Association of human papillomavirus and p16 status with
Wang	Kantoff	J Clin Oncol	17.879	133 258	Association of SLCO2B1 genotypes with time to progression
Xing	Sykorova	J Clin Oncol	17.879	133 258	Association between BRAF V600E mutation and recurrence
Yu	Thompson	J Clin Oncol	17.879	133 258	SWOG S0925: a randomized phase II study of androgen
Liu	Fyles	J Clin Oncol	17.879	133 258	Identification of a low-risk luminal a breast canc
Fang	Lee	J Clin Oncol	17.879	133 258	C-reactive protein as a marker of melanoma progression
Goldman	Pollack	J Clin Oncol	17.879	133 258	Phase II trial assessing the ability of neoadjuvant
Ahearn	Lotan	JNCI	15.161	36 458	A prospective investigation of PTEN loss and ERG
Carvajal-Hausdorf	Rimm	JNCI	15.161	36 458	Measurement of domain-specific HER2 (ERBB2)
Goldstein	Von Hoff	JNCI	15.161	36 458	Nab-paclitaxel plus gemcitabine for metastatic
Gupta	Tsao	JNCI	15.161	36 458	Gender Disparity and Mutation Burden in Metastatic
Hall	Lucci	JNCI	15.161	36 458	Circulating Tumor Cells and Recurrence After

Hur	Goel	JNCI	15.161	36 458	Identification of a Metastasis-Specific MicroRNA
Sahm	von Deimling	JNCI	15.161	36 458	TERT Promoter Mutations and Risk of Recurrence in Meningioma
Scurr	Godkin	JNCI	15.161	36 458	Assessing the Prognostic Value of Preoperative
Saiag	Boniol	JNCI	15.161	36 458	Prognostic Value of 25-hydroxyvitamin D3 Levels
Cremolini	Falcone	Lancet Oncol	24.725	24 861	FOLFOXIRI plus bevacizumab versus FOLFIRI plus
Cybulski	Lubiński	Lancet Oncol	24.725	24 861	Clinical outcomes in women with breast cancer and a PALB2
Tabernero	Van Cutsem	Lancet Oncol	24.725	24 861	Analysis of circulating DNA and protein biomarkers to
Torchia	Huang	Lancet Oncol	24.725	24 861	Molecular subgroups of atypical teratoid rhabdoid

CLINICAL FACTORS

Copson	Eccles	Ann Oncol	6.578	26807	Obesity and the outcome of young breast cancer patients in the UK
Cremolini	Falcone	Ann Oncol	6.578	26807	Early tumor shrinkage and depth of response predict long-term outcome
Jiang	Vickers	Ann Oncol	6.578	26807	Clinical outcomes of elderly patients receiving neoadjuvant chemoradiation
Sun	Park	Ann Oncol	6.578	26807	Small-cell lung cancer detection in never-smokers: clinical characteristics
Sogaard	Sorensen	Blood	9.775	150 854	Splanchnic venous thrombosis is a marker of cancer and a prognostic
Ellingson	Cloughesy	Clin Cancer Res	8.193	72 155	Quantification of nonenhancing tumor Burden in
Wang	Li	Clin Cancer Res	8.193	72 155	The predictive and prognostic value of early
Gershenson	Sun	J Clin Oncol	17.879	133 258	Impact of age and primary disease site on outcome in
Johung	Contessa	J Clin Oncol	17.879	133 258	Extended survival and prognostic factors for patients
Klotz	Loblaw	J Clin Oncol	17.879	133 258	Long-term follow-up of a large active surveillance
Lauritsen	Daugaard	J Clin Oncol	17.879	133 258	Germ cell cancer and multiple relapses: toxicity
Martin	Baracos	J Clin Oncol	17.879	133 258	Diagnostic criteria for the classification of
Renfro	Lenz	J Clin Oncol	17.879	133 258	Body mass index is prognostic in metastatic colorectal
Tsao	Brambilla	J Clin Oncol	17.879	133 258	Subtype classification of lung adenocarcinoma predicts

Wikman	Lagergren	J Clin Oncol	17.879	133 258	Psychiatric morbidity and survival after surgery for
Yang	Campbell	J Clin Oncol	17.879	133 258	Active smoking and mortality among colorectal cancer
Yuan	Wolpin	J Clin Oncol	17.879	133 258	Survival among patients with pancreatic cancer and long-
Haque	Kwan	JNCI	15.161	36 458	Tamoxifen and antidepressant drug interaction in a
Hoffmeister	Brenner	JNCI	15.161	36 458	Statin use and survival after colorectal cancer: the
Kwan	Caan	JNCI	15.161	36 458	Breastfeeding, PAM50 tumor subtype, and breast
Ligibel	Barry	JNCI	15.161	36 458	Body mass index, PAM50 subtype, and outcomes in
Loupakis	Lenz	JNCI	15.161	36 458	Primary tumor location as a prognostic factor in
Nyante	Gierach	JNCI	15.161	36 458	Prognostic significance of mammographic density
Ohri	Werner-Wasik	JNCI	15.161	36 458	Pretreatment FDG-PET metrics in stage III non-small
Yoon	Sinicrope	JNCI	15.161	36 458	Racial differences in BRAF/KRAS mutation rates and
