# Supplementary material for: Overinterpretation and misreporting of prognostic factor studies in oncology: a systematic review
Source: Br J Cancer. 2018 Oct 24;119(10):1288–96. doi: 10.1038/s41416-018-0305-5 (PMC6251031; doi:10.1038/s41416-018-0305-5)
Supplement: Supplementary file 4 — Supplementary Information [file 41416_2018_305_MOESM4_ESM.pdf]

REPORT CHARACTERISTICS

Article ID …………………………. **Reader ID** ………………………….

Academic position of the 1st author as reported:

☐ senior researcher (PhD) ☐ junior researcher (MD, MSc) ☐ Not reported

Scientific background of the 1st author: ☐ clinician ☐ biologist

Is **any statistician or epidemiologist reported clearly** among the co-authors?

☐ Yes ☐ No ☐Not reported

Funding source: ☐None ☐Profit ☐Non-profit ☐Both ☐Not reported

Is there a "Disclosure of authors' potential **conflicts of interest** (COI)" section? ☐ Yes ☐ No

Are COI reported? ☐ Yes ☐ No

THE ARTICLE

REPORT – METHODS SECTION

The aim of the original patient dataset creation was an ☐ Interventional study

☐ Observational study

The outcome assessment is prospective ☐ Yes ☐ No

The PF assessment is prospective ☐ Yes ☐ No

Mention of adherence to **REMARK** guidelines ☐ Yes ☐ No

How many different populations were used to assess PF effect (eg, exploratory and confirmatory samples ⇔ 2, 3 trial populations merged ⇔ 1)? ……………………………………………………

If >1, please focus on the biggest population for further extraction

If >1, does the validation dataset originate from the same one than the exploratory dataset does?

☐ Yes ☐ No

Are the variables **prespecified** before any statistical analysis? ☐ Yes ☐ No

Is the method of how **missing data** are handled reported? ☐ Yes ☐ No

Is the method of how **continuous data** are handled reported/ justified? ☐ Yes ☐ No

Are the methods of **variable selection procedures** reported? ☐ Yes ☐ No

Is any **subgroup analysis prespecified**? ☐ Yes ☐ No

If yes, **how many subgroup criteria** are defined? …………………………………………

Is any **sensitivity / subpopulation** analysis prespecified? ☐ Yes ☐ No

If yes, **how many subpopulation criteria** are defined?……………………………………………….

How many multivariable models are prespecified, if any? ………………………………………………

REPORT - RESULTS SECTION

Sample size …….. **Number of events per outcome**………..**Median follow-up length** (mo)………..

Number of outcomes ……………………………………………………………………………….

Type of outcomes: overall survival, progression-free survival, disease-free survival, time-to-progression, other (If other, please specify …………………………………)

Number of PFs ………………………………………………………………………………………

Type of PFs: please specify……………………………………………………………………………..

Number of PF- outcome associations reported ……………………..… (**excluding subgroup analyses**)

Reporting of result for PF-outcome associations (if many, choose the best option)

- ☐ Kaplan-Meier curve outcomes with statistical comparison (logrank test)
- ☐ Kaplan-Meier curve outcomes without statistical comparison
- ☐ Univariate analysis: ☐ Estimated effect size and its precision
  - ☐ Estimated effect size without precision reported
  - ☐ Only p-value
- ☐ Multivariable analysis ☐ Estimated effect size and its precision
  - ☐ Estimated effect size without precision reported
  - ☐ Only p-value
- ☐ Using generic statement only
- ☐ Other statistical analysis (i.e. Pearson correlation …)
- ☐ Not reported

Is there **any heterogeneity in the way PF-outcome associations** are reported? ☐ Yes ☐ No

Is there any incomplete reporting? ☐ Yes ☐ No

Number of adjusted multivariable models reported per PF effect (if any) **(defined by the type of the variables of adjustment)** ……….

Are the PF-outcome associations reported in **supplemental files**? ☐ Yes ☐ No

Is any **association between other variables / confounders and the outcome missing (only in multivariable model)?** ☐ Yes ☐ No ☐ Not applicable (NA)

Is at least ONE non-significant association among all the PF – outcome pairs (for all the subclasses of the categorization) derived **from a multivariable model** reported? ☐ Yes ☐ No

Is the PF effect adjusted for **multiple comparisons**? ☐ Yes ☐ No

Reporting of **association between PF and other variables of the model** ☐ Yes ☐ No

Is any **association between other variables / confounders and the outcome missing (only in multivariable model)?** ☐ Yes ☐ No ☐ Not applicable (NA)

Linguistic spin ☐ Yes ☐ No

If yes: Use of **leading words to reject non statistically significance** / trend ☐

Use of **strong "statement"** for PF prognostic value ☐

Other………………………………………. ☐

Use of ☐ "statistically significant"

☐ "significant" without "statistically"

☐ none

Is there **any subgroup analysis** reported? ☐ Yes ☐ No

If yes,

Is their reporting consistent with what was prespecified? ☐ Yes ☐ No

How many **subgroup criteria are reported?** ………..……………………….

All in all, how many **PF-outcome associations are reported within the subgroup analysis?** ……………………………………………………………..

Is there **any interaction test** *p*-value reported? ☐ Yes ☐ No

Is there **any subpopulation** or sensitivity analysis reported? ☐ Yes ☐ No

Is their reporting consistent with what was prespecified? ☐ Yes ☐ No

How many **subpopulations are reported?** ………………………………..

How many **PF-outcome associations are reported?** ………………………

How many statistical tests were performed in respect of PF effect assessment (including baseline patient characteristics, including subgroup and sensitivity analysis, and <u>**Supplemental files**</u>) ……………………………..

Spin in the presentation of tables and figures ☐ Yes ☐ No

Is the PF defined in multiple ways? (eg, different thresholds of categorization AND continuous, or absolute value and relative value, …) ☐ Yes ☐ No

Are continuous data reported as **categorized or dichotomized**? ☐ Yes ☐ No

Other misleading strategies ☐ Yes ☐ No

If yes, detail ……………………………………………………

REPORT - DISCUSSION SECTION

Linguistic spin

Use of **strong "statement"** <u>for PF effectiveness</u> ☐ Yes ☐ No

Use of **leading words** <u>to reject/ explain non-statistically significance / trend</u> ☐ Yes ☐ No

Other……………………………………………. ☐ Yes ☐ No

Limitations reported ☐ Yes ☐ No

References to other published studies assessing the PF effect

<u>Systematic review or meta-analyses or trials reported</u>

in favor of the PF ☐ Yes ☐ No

not in favor of the PF ☐ Yes ☐ No

in favor of some outcomes and not in favor for others ☐ Yes ☐ No

CONCLUSION SECTION OR LAST PARAGRAPH OF THE DISCUSSION SECTION

Mention of any prognostic value of PF ☐**Yes, positive** ☐**Yes, negative** ☐ **No**

Consistent with study results (regardless of the type of statistical analysis used) ☐**Yes** ☐ **No**

Focus solely on significant results (absence of NS results reports) ☐**Yes** ☐ **No**

Report perspectives ☐**Yes** ☐ **No**

Use the PF in clinical practice or clinical research (trial design) ☐ Yes ☐ No

If yes, is it into a different clinical setting or population? ☐ Yes ☐ No

Recommendations for further PF assessment study ☐ Yes ☐ No

Other perspectives ☐ Yes ☐ No

Uncertainty reported ☐ Yes ☐ No

Linguistic spin ☐**Yes** ☐ **No**

If yes detail……………………………..…………….……………….………………..……

Other misleading strategy ☐**Yes** ☐ **No**

If yes detail……………………………..…………….……………….………………..……

ABSTRACT – RESULTS SECTION

Is at least one outcome related to PF evaluation clearly identified in the abstract? (e.g. "overall survival" but not "clinical benefit") ☐ Yes ☐ No

Number of outcomes which are mentioned ……………………………………………………………..

Number of PFs which are mentioned …………………………………………………………………

Number of outcome – PF associations which are reported …..…………………………………………..

Reporting of result for each PF-outcome pair (1st, 2nd, 3rd …):

☐ Kaplan-Meier (KM) analysis with statistical comparison (<u>i.e. logrank test)</u>

☐ KM results <u>without</u> statistical comparison (i.e. survival in each PF group)

☐ Univariate analysis: ☐ Estimated effect size and its precision

☐ Estimated effect size <u>without</u> precision reported

☐ Multivariable analysis ☐ Estimated effect size and its precision

☐ Estimated effect size <u>without</u> precision reported

☐ Using generic statement only

☐ Not reported

☐ Other……………………….

Heterogeneity in PF-outcome associations reporting ☐ Yes ☐ No ☐ NA

**Linguistic spin**	☐ Yes ☐ No
Strong statement	☐ Yes ☐ No
Emphasis on PF clinical relevance while NS (trend / reject NS)	☐ Yes ☐ No
Other	☐ Yes ☐ No
**Reporting of subgroup / subpopulation analysis**	☐ Yes ☐ No
If yes, was it **prespecified?**	☐ Yes ☐ No
If yes, was subgroup analysis reported with **interaction test p-value?**	☐ Yes ☐ No
**Other misleading strategies**	☐ Yes ☐ No

If yes, detail ……………………………………………………..

ABSTRACT – CONCLUSION SECTION

**Mention of any PF prognostic value**	☐**Yes, negative** ☐**Yes, positive** ☐ **No**
**Consistent with study results** (regardless of the type of statistical analysis used)	☐**Yes** ☐ **No**
**Linguistic spin**	☐ **Yes** ☐ **No**
Emphasis on PF clinical relevance while NS (trend / reject)	☐ Yes ☐ No
Strong statement	☐ Yes ☐ No
Other	☐ Yes ☐ No
**Perspectives**	☐**Yes** ☐ **No**
PF use in clinical practice	☐ Yes ☐ No
If yes, other setting	☐ Yes ☐ No
Further study for PF assessment	☐ Yes ☐ No
Other perspective (i.e., predictiveness, other biomarkers …)	☐ Yes ☐ No
**Other misleading strategies**	☐**Yes** ☐ **No**

If yes detail……………………………..……………….……………….………………..……

**Limitations**	☐ Yes ☐ No
**Uncertainty**	☐ Yes ☐ No

TITLE

Supportive of the clinical relevance of the PF? ☐ Yes ☐ No
Is that irrelevant? ☐ Yes ☐ No

COMMENTS
